# Simulation-free magnetic resonance-guided radiation therapy of prostate cancer

**DOI:** 10.1016/j.phro.2024.100667

**Published:** 2024-11-07

**Authors:** Cora Warda, Cihan Gani, Simon Boeke, David Mönnich, Moritz Schneider, Maximilian Niyazi, Daniela Thorwarth

**Affiliations:** a Section for Biomedical Physics, Department of Radiation Oncology, University Hospital Tübingen, Tübingen, Germany; b German Cancer Consortium (DKTK), Partner Site Tübingen, a Partnership Between DKFZ and University Hospital Tübingen, Germany; c Department of Radiation Oncology, University Hospital Tübingen, Tübingen, Germany

**Keywords:** MRI-Linac, Planning study, Diagnostic CT data, Online adaptive MRI-guided radiation therapy, Simulation-free planning

## Abstract

**Background and purpose:**

Despite recent advances of online image-guided high-precision patient positioning and adaptation using magnetic resonance imaging (MRI) or cone-beam computed tomography (CT), standard radiation therapy pathway still involves a dedicated simulation scan. The aim of this study was to evaluate the feasibility and planning quality of integrating a simulation-free treatment planning workflow for adaptive online MRI-guided radiation therapy on a 1.5 T MRI linear accelerator (MRI-Linac) in prostate cancer using diagnostic CT (dCT) scans.

**Materials and methods:**

For ten patients with prostate cancer previously treated at the MRI-Linac with adaptive radiation therapy (42.7 Gy in 7 fractions), simulation-free reference plans based on dCT were retrospectively created, and adaptive plans were simulated for the first treatment fraction. Reference and adapted plans derived from both standard and simulation-free workflows were compared with regard to institutional dose/volume criteria, followed by statistical assessment using the paired Wilcoxon signed-rank test with a Bonferroni-corrected significance level of α = 0.025.

**Results:**

Simulation-free reference and adapted plans consistently met dose/volume criteria. Statistical analysis revealed no significant differences between both workflows, except median values for near-maximum dose (D2%) in the planning target volume: 44.2 Gy (standard) vs. 44.5 Gy (simulation-free) in reference plans (p = 0.01), and 44.5 Gy vs. 44.6 Gy in adapted plans (p = 0.01).

**Conclusion:**

This study demonstrated the feasibility of simulation-free radiation therapy planning using dCT. Comparable treatment plan quality was observed for both reference and adapted radiation therapy plans in a curative setting for patients with prostate cancer.

## 1 Introduction

Over the past decade, advancements in technology and interdisciplinary research within the clinical setting have brought radiation therapy (RT) to an ever-higher degree of precision [1]. The advent of image-guided RT and the further development of adaptive RT enable real-time adjustments to take account of shifts in the patient's position, organ movements, as well as changes in tumor position and volume [2], [3], [4], [5], [6]. Despite the wide range of options for precise positioning and the recalculation and adaptation of treatment plans according to the daily situation, the current clinical standard procedure for plan preparation including highly reproducible positioning foresees a simulation session consisting of a dedicated RT planning computed tomography (pCT) or planning magnetic resonance imaging (pMRI) scan.

Previous studies have shown that RT planning based on existing diagnostic imaging data and omission of pCT in palliative treatment situations is possible within a simulation-free workflow [7], [8], [9]. The ability to improve positioning accuracy and the possibility of online adaptation using on-board imaging such as cone-beam CT (CBCT) or MRI have enabled simulation-free RT of bone and soft tissue metastases in regions such as the spine, thorax or abdomen [7], [8], [10], [11]. In the study by Price et al. [12], a simulation-free workflow at an integrated adaptive O-Ring gantry system was implemented, where the target volume was defined in a pre-plan using a diagnostic CT (dCT). The integration of artificial intelligence recently demonstrated the possibility of generating pCT scans from dCT, CBCT or MRI streamlining and improving the workflow in RT [13], [14], [15].

As further studies have shown, the use of population-based electron density data provides results comparable to the use of electron densities from a gold standard or pCT [16], [17]. The combination of population-based electron density data and online plan adaption, taking account for changes in patient positioning, could now enable to use dCT data as a simulation scan, including contouring, target volume definition, and dose calculation further streamlining the RT workflow.

With the elimination of the simulation scan, a shorter overall treatment time can be realized and hospital resources in terms of personnel and equipment can be spared [18], [19], [20], [21], [22]. From the previously published studies, several recurring challenges in implementing the simulation-free workflow have become apparent.

These challenges encompassed issues related to consistent patient positioning, field of view (FOV), the use of various tabletops, respiratory motion management and the calibration of electron density assignments for dCT scanners [9], [11], [17], [22], [23], [24], [25]. Hybrid systems combining a linear accelerator (Linac) and an imaging modality with the option of online adaptive radiotherapy seem to be an optimal technology for the implementation of simulation-free workflows. The study by De Leon et al. presented initial results of a simulation-free workflow at the magnetic resonance imaging linear accelerator (MRI-Linac) for stereotactic ablative body radiotherapy in curative treatment, comparing reference plans based on prostate specific membrane antigen positron emission tomography CTs (PSMA-PET/ CT) with those derived from pCTs [26]. Re-imaging for each treatment fraction allows plan adaptation to the real-time position of the planning target volume (PTV) and organs at risk (OAR). The online adaptive workflow at the MRI-Linac, based on daily MRI datasets, is one option that can help to overcome the key challenges related to a simulation-free workflow targeting also curative treatment approaches.

With regard to curative treatments, prostate carcinoma may be suitable for a simulation-free study, as the same anatomical region is consistently treated, and planning is conducted according to a standardized protocol, including target volume definition and margin recipe.

Therefore, the aim of this study was to assess the feasibility of simulation-free treatment planning approach for online adaptive MRI-guided radiation therapy (MRIgRT) based on various dCT data. And the investigation of plan quality of reference and adapted plans compared to conventional MRI-only planning with dedicated pMRI for patients with prostate cancer (PC).

## 2 Materials and methods

### 2.1 Patients and imaging

A total of ten patients with low or intermediate risk PC who were previously treated with 42.7 Gy in seven fractions at the 1.5 T MRI-Linac (Unity, Elekta AB, Sweden) between 09/2021 and 04/2023 were included in this study. All patients had a dedicated pMRI and four cases also had a pCT scan. These pCT scans were not utilized for this study in order to make the study setup consistent. A dCT was available for all patients before the start of RT. dCT scans were taken from the clinical data base and may therefore present with varying scan protocols, cf. Table 1. The study was part of a prospective trial which was approved by the local ethical committee (659/2017BO1, NCT04172753). All patients gave written informed consent.

**Table 1:** Overview of the patient characteristics and image properties of the acquitted dCTs. The resolution of the CT images is indicated by the pixel spacing and the size of the FOV.

Patient	Age [y]	TNM- classifi-cation [29]	CT con-trast agent	Time between dCT and start of RT [d]	X-ray tube voltage [kVp]	x-/y- Pixel spacing [mm]	FOV [cm^2^]
P01	69	T2c N0 M0	Yes	124	90	0.56	28.8 × 37.8
P02	73	T2a N0 M0	Yes	84	90	0.53	27.3 × 36.6
P03	65	T2c N0 M0	Yes	48	100	0.89	45.7 × 45.7
P04	73	T2a N0 M0	Yes	58	100	0.87	44.4 × 44.3
P05	67	T2a N0 M0	No	113	130	0.82	41.9 × 41.9
P06	76	T2b N0 M1	No	67	130	0.80	40.8 × 40.8
P07	75	T2c N0 MX	Yes	26	100	0.89	45.6 × 45.6
P08	79	T2a N0 M0	No	42	120	0.98	49.9 × 49.9
P09	80	T2c N0 M0	Yes	117	120	0.84	42.8 × 42.8
P10	77	T1c N0 M0	No	15	120	0.98	49.9 × 49.9

### 2.2 Standard reference planning

Standard reference plans were created according to our standardized institutional PC RT simulation and treatment planning workflow. This procedure encompassed a pMRI for all patients. MRI-only planning was used for all patients. For both simulation scans, patients were positioned on a flat tabletop with indexed knee rolls and feet securely fixed. Contouring was performed automatically using a commercial software (ARTplan, TheraPanacea, France), supplemented by further manual refinement of the target volume and a comprehensive review of all anatomical structures by a board-certified radiation oncologist. The PTV was established using a consistent margin approach where the segmented prostate structure was expanded by 6 mm in the left, right, superior, inferior, and anterior directions, and by 5 mm in the posterior direction. Following this, treatment planning and dose calculation were carried out using Monaco 5.51.11 (Elekta AB, Sweden). The plans were generated based on a standardized planning template, which included nine beam angles (0°, 30°, 60°, 100°, 135°, 225°, 260°, 300°, 330°), standardized set of cost functions, along with data on the electron density and structure set of couch and coils of the MRI-Linac.

A population-based bulk density approach [16] was used to assign electron densities to the structures of the femoral heads, pelvis, sacrum, bladder, rectum and patient for MRI-only planning.

### 2.3 Simulation-free reference planning

The dCTs used were gathered from the hospital database including abdominal and whole body CT protocols, as well as positron emission tomography CTs. If dCTs were acquired in a feet-first supine orientation, a coordinate transformation was performed to achieve a head-first supine orientation in the planning system. The dCT FOV had to cover the target volume, the external body contour, and all relevant OARs. If there were several dCT datasets, the most recent one was used, with no more than one year between the acquisition of the diagnostic image dataset and the treatment cf. Table 1 for detailed information. In accordance with the standard reference planning workflow, contouring was conducted automatically using the commercial software ART-plan. The PTV was established using the same consistent margin approach as in the standard workflow. For simulation-free RT planning, a standardized planning template using the same gantry angles and the same population-based bulk density approach as in the standard reference plans [16] was used.

### 2.4 Standard plan adaptation

Adaptation of the standard reference plan was based on the daily MRI scan of the first fraction. Delineated structures from the reference plan were propagated rigidly and deformable to the acquired MRI and the electron density information from the population-based bulk density approach was integrated. A radiation oncologist manually adapted the structure delineation to match the current anatomy. As part of the online adaptation process, the reference plan was reoptimized using the adapted structure set and the Monaco treatment planning software. If necessary, the planner adjusted the cost functions during plan re-optimization to meet institutional dose/volume criteria (IDVC).

### 2.5 Simulation-free plan adaptation

MRI-guided plan adaptation of the simulation-free reference plans was simulated mimicking a clinical situation on the MRI-Linac. Therefore, the simulation-free reference plans were adapted using the same daily MRI dataset including structure set, which had been adjusted by the radiation oncologist during MRIgRT treatment of the first fraction. The simulation-free reference plan was adapted and re-optimized based on the MRI data set of the day in the adaptive workflow. Minor adjustments to the cost function settings were made manually during the re-optimization of the plan, if needed.

### 2.6 Data analysis

Plan quality was assessed comparing standard and simulation-free reference as well as adapted plans based on our IDVC for plan acceptance (Table 2). The simulation-free adapted plans were evaluated using the same MRI dataset, including structure set, that was also used for the standard treatment pathway in the first adaptive fraction. Additionally, the numbers of segments and monitor units (MU) were determined for each set of treatment plans. The statistical analysis was conducted in MATLAB R2023a using a paired Wilcoxon signed rank-test with a significance level of 0.025 (0.05/2 = 0.025), Bonferroni corrected for multiple testing.

**Table 2:** Institutional dose/volume criteria (IDVC) for patients with prostate cancer (42.7 Gy in seven fractions) and corresponding statistical test results between pCT and dCT plans regarding reference and adapted plans. Statistically significant values are indicated in bold letters.

Structure	IDVC	p-value Reference Adapted	
Clinical target volume	D98% > 40.6 Gy	0.56	0.38
Planning target volume	D2% < 45.7 Gy	**0.01**	**0.01**
Planning target volume	D98% > 38.4 Gy	0.38	0.70
Rectum	V42.7 Gy < 0.01 %	1	1
Rectum	V38.4 Gy < 15.0 %	0.05	0.07
Rectum	V32.0 Gy < 35.0 %	0.16	0.36
Rectum	V28.0 Gy < 45.0 %	0.24	0.85
Urethra	D0.05 cm^3^ < 42.7 Gy	0.51	0.70
Bladder	V42.7 Gy < 2 cm^3^	0.77	0.04

## 3 Results

The mean time delay between dCT and the start of RT was 69.4 days, with a standard deviation of 38.8 days.

### 3.1 Comparison of reference plans

**Fig. 1:**
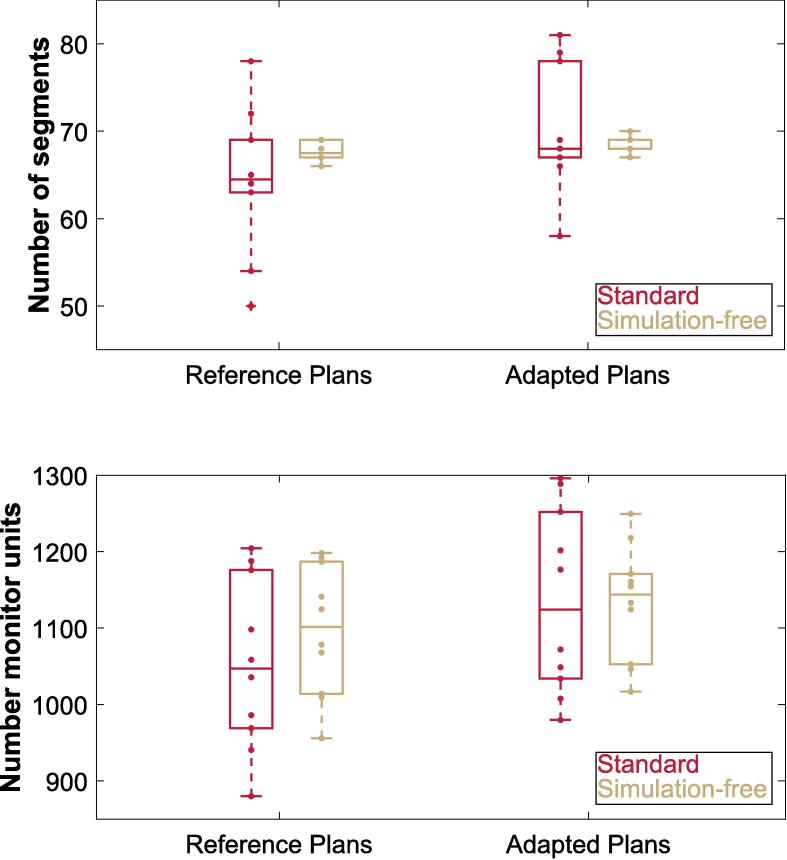
Number of segments (top row) and number of monitor units (bottom row) per treatment plan, for reference plans and adapted plans. The standard plans are depicted in red, while the simulation-free plans are illustrated in gold. Outliers are depicted with crosses. (For interpretation of the references to color in this figure legend, the reader is referred to the web version of this article.)

In terms of plan complexity, no significant differences were observed regarding the total number of MU and segments. The number of segments in the reference plans exhibited a median (range) of 64.5 (50–78) for the standard plans and 67.5 (66–69) for the simulation-free plans (p = ns). Fig. 1 depicts the number of segments and MU per treatment plan of standard and simulation-free workflows. Similarly, the median number of MU was not significantly different between standard and simulation-free reference plans with 1047 MU (880–1204 MU) and 1101 MU (956–1198 MU), respectively (p = ns, Fig. 1). Both, standard and simulation-free reference plans met all required IDVC as specified in Table 2.

**Fig. 2:**
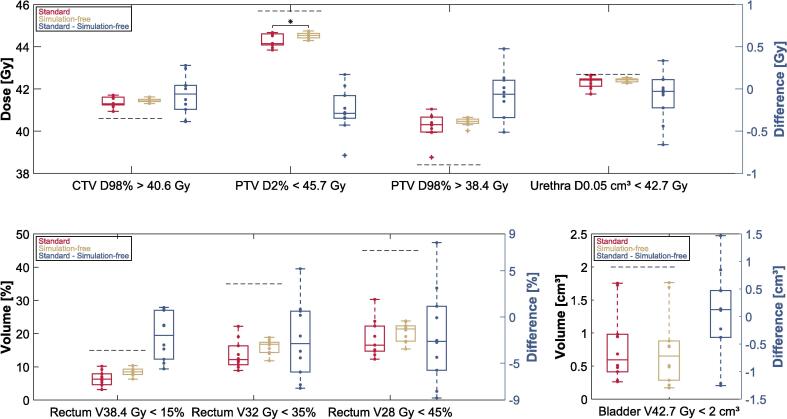
Comparison of the standard (red) and simulation-free (gold) reference plans. Boxplots with differences were determined by subtracting the results of the simulation-free workflow from those of the standard workflow. Institutional dose/volume criteria (IDVC) for planning are represented with grey dashed lines, while individual values are depicted as dots in the corresponding color and outliers are identified with crosses. Significant results (p < 0.025, paired Wilcoxon signed rank-test) are marked with *. (For interpretation of the references to color in this figure legend, the reader is referred to the web version of this article.)

Direct comparison of individual IDVC in the standard and simulation-free reference plans revealed a median (range) of clinical target volume (CTV) near-minimum dose (D98%) of 41.3 Gy (40.9–41.7 Gy) for the standard plans and 41.4 Gy (41.3–41.6 Gy) for the simulation-free plans (p = ns, Fig. 2). For PTV D98%, median doses of 40.3 Gy (38.7–41.1 Gy) and 40.5 Gy (40.0–40.7 Gy) were achieved (p = ns).

A significant difference was found for PTV D2% where the standard reference plans had a median value of 44.2 Gy (43.8 – 44.7 Gy), while the simulation-free plans achieved 44.5 Gy (44.3 – 44.8 Gy), p = 0.01. As shown in Fig. 2, no significant differences were observed for all other IDVC. All reference plans from both the standard and simulation-free workflows met the maximum dose criteria for the rectum (V42.7 Gy < 0.01 %). In the simulation-free workflow, nine cases had a value of 0 % and one case had a value < 0.01 %. In the standard plans, eight cases had a value of 0 % and two cases had a value < 0.01 %.

### 3.2 Comparison of adapted plans

Regarding the evaluation of MU and segments of adapted standard and simulation-free plans, no significant differences were found (p = ns). The standard adapted plans had a median (range) of 68 (58–81) segments per treatment plan, whereas the simulation-free adapted plans showed 69 (67–70) segments (p = ns, Fig. 1). In terms of MU, the standard plans presented with values of 1124 MU (980–1279 MU), while the simulation-free plans showed values of 1144 MU (1017–1250 MU), (p = ns, Fig. 1).

**Fig. 3:**
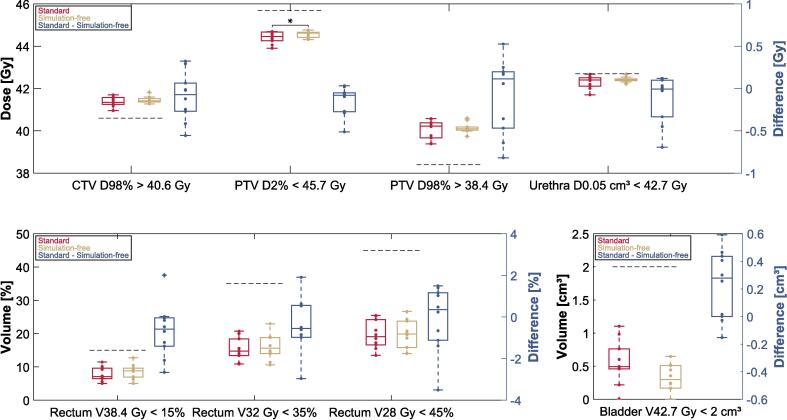
Comparison of the adapted plans between the standard (red) and simulation-free (gold) approach. Boxplots with differences were determined by subtracting the results of the simulation-free workflow from those of the standard workflow. The IDVC are represented with grey dashed lines, individual values of the patients are depicted as dots and outliers are identified with crosses. Significant results of paired Wilcoxon signed rank-test are marked with * (p < 0.025). (For interpretation of the references to color in this figure legend, the reader is referred to the web version of this article.)

All standard and simulation-free adapted plans successfully achieved the IDVC requirements. The evaluation of the adapted plans showed a median (range) CTV D98% of 41.4 Gy (41.0–41.7 Gy) for the standard plans and 41.4 Gy (41.3– 41.8 Gy) for the simulation-free plans (p = ns). Regarding the PTV D98%, the median dose was in 40.2 Gy (39.4–40.6 Gy) for the standard plans and 40.1 Gy (39.7–40.6 Gy) for the simulation-free plans (p = ns). A significant difference was found for PTV criteria, as shown in detail in Fig. 3. The standard plans showed median values of the PTV D2% of 44.5 Gy (43.9–44.7 Gy) and the simulation-free plans of 44.6 Gy (44.3–44.7 Gy) (p = 0.01). All other IDVC did not show any significant differences in the evaluation. The absolute differences in the IDVC of the rectum and bladder between the two workflows seem smaller in the adapted plans compared to the reference plans, cf. Fig. 2 and Fig. 3. Both, the plans from the standard and simulation-free workflow, met the maximum dose criteria for the rectum (V42.7 Gy < 0.01 %). Specifically, there were eight cases each in the simulation-free and standard plans with a value of 0 % and two cases each with a value < 0.01 %.

**Fig. 4:**
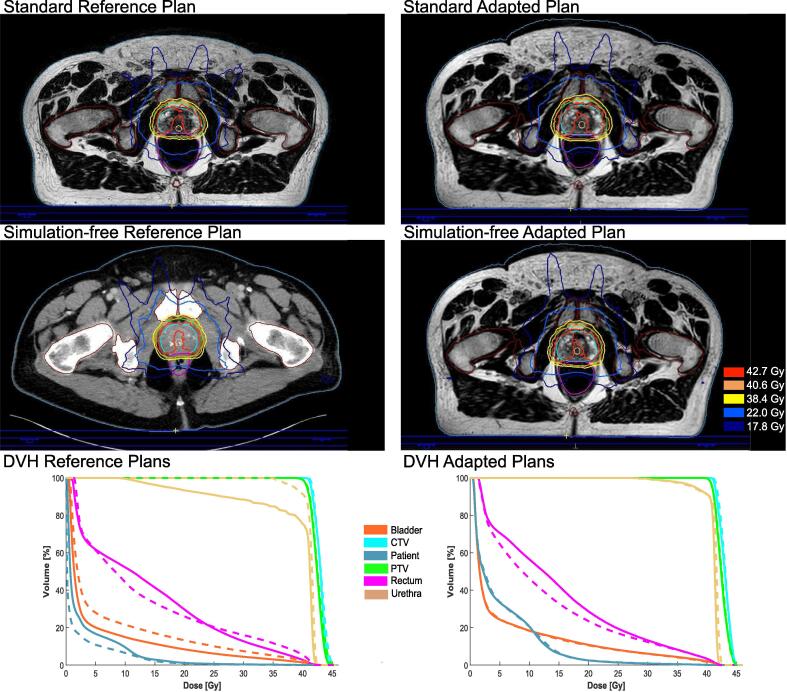
Exemplary images of patient #4 dose distribution of the standard reference plan (top left), standard adapted plan (top right), simulation-free reference plan (middle left), simulation-free adapted plan (middle right) and DVHs of both reference plans (bottom left) and both adapted plans (bottom right). Solid lines indicate the standard plans and dashed lines the simulation-free plans.

Fig. 4 exemplarily shows reference and adapted plans created based on pMRI and dCT, of patient #4 and the according dose volume histograms (DVHs).

## 4 Discussion

In this retrospective study, dCT scans were utilized for simulation-free treatment planning using online adaptive MRIgRT on the 1.5 T MRI-Linac and compared to the standard pMRI-based simulation workflow. The feasibility of this simulation-free approach employing dCTs for planning was substantiated by comparable treatment plan quality observed for both, reference and adapted RT plans, in a cohort of patients with PC.

The primary objective of this study was to explore the feasibility of generating clinically viable treatment plans using dCT images, which could then be adapted to the specific conditions of the first fraction on the 1.5 T MRI-Linac. Prior studies, such as Fakhoury et al. [11], focused on optimizing emergency RT procedures with an emphasis on achieving reproducibility in patient positioning. Deviations due to table shape, and arm position of the patient could be adapted by a protocol for performing dCTs [27]. This study, using online adaptive MRIgRT, demonstrated the ability to compensate for anatomical and technical discrepancies between dCT scans and the actual irradiation scenario. The presented method of a simulation-free treatment approach can also be applied to other adaptive workflows, such as CBCT-guided online radiotherapy, also confirmed by a recent study [12].

Similar to the findings in the study by Mittauer et al. [7], the adaptation process benefited from the high soft tissue contrast provided by the daily MRI. By including low or intermediate risk patients without additional hormone therapy with a time interval between diagnosis and treatment of approximately ten weeks in contrast to studies by Nelissen et al. [8], [10], the approach might enable to provide insights regarding treatment planning and dose distribution well in advance of the actual treatment. Furthermore, we demonstrated that the online plan adaptation is also feasible for patients with dCT acquired more than 100 days before.

This study has some limitations that warrant consideration. The study exclusively utilized dCT data, excluding the incorporation of diagnostic MRI datasets. Previous research has demonstrated that by including MRI, an MRI-only workflow can be established, thus eliminating the need for planning on CT imaging data [11], [26], [27]. The dCT datasets included in this study were obtained using different CT scan protocols, such as liver, thorax-abdomen, and skeletal scintigraphy, which resulted in a variation regarding scan direction, pixel spacing, and FOV size in our diagnostic datasets. This initial study tested the simulation-free workflow only on patients with PC. Provided further results, there seems to be potential to translate this workflow to other tumor entities. The results indicate significant differences between the standard and simulation-free plans in the IDVC for the PTV in both the reference and adapted plans, despite the significance both approaches resulted in absolute IDVC values far below our institutional planning objectives.

Possible explanations for the trend toward higher IDVC values in the simulation-free reference plans for the bladder and rectum, compared to the standard reference plans, cf. Fig. 2, might be the lack of specific patient preparation for bladder and rectum filling, as no drinking protocols or rectum filling controls were provided in the dCTs. Another factor might be that the standard plans were created in a clinical setting by multiple medical physicists and radiotherapy technologists, whereas the simulation-free plans were generated by a single operator. Our results for the simulation-free adapted plans demonstrate that it is possible to successfully adapt these plans within the adaptive online MRIgRT workflow. This is further supported by a much smaller difference between simulation-free and standard adapted plans regarding IDVC values for the rectum and bladder (Fig. 3). Despite these trends, the simulation-free reference and adapted plans consistently met all IDVC.

The non-significant variations in the number of segments and MU as well as the specifications pertaining to individual anatomical structures can be elucidated by the planner-specific preferences from multiple operators in the standard workflow and one operator in the simulation-free workflow during the creation and manual optimization of the plans. In the reference plans, discrepancies in contour delineation may be due to the use of an automatic contouring procedure.

In contrast to previous studies, our study demonstrated adherence to clinical guidelines regarding target coverage and OAR sparing for reference and adapted plans in a curative setting. While Nelissen et al. [8] achieved differences of less than 0.2 % in target volume coverage (V95%) between reference plans and adapted plans, the results of Kejda et al. [28] showed a mean coverage of the CTV and PTV (V95%) of 99.5 % and 99.2 % respectively. This study also attained compliant coverage of CTVs and PTVs in both the reference plans and adapted plans within the standard and simulation-free workflow, aligning with our IDVC.

To conclude, the simulation-free workflow involving dCT-based treatment planning and adaptation during online adaptive MRIgRT on the 1.5 T MRI-Linac is feasible and yields outcomes comparable to the existing standard workflow, with a dedicated simulation scan. The specific patient positioning, FOV adjustments, changes in organs, and variations in table shape, present in the dCT, can all be effectively compensated for through online adaptation in this simulation-free procedure. Moreover, exploration of additional tumor entities with curative treatment objective should be evaluated to assess the potential for implementing simulation-free planning to increase the generalizability of the simulation-free approach.

## 5 Financial support

This study received funding by the German Research Council (DFG Grants No. ZI 736/2-1).

## Declaration of competing interest

The authors declare that they have no known competing financial interests or personal relationships that could have appeared to influence the work reported in this paper.

## References

[1] Baumann M., Krause M., Overgaard J., Debus J., Bentzen S.M., Daartz J. (2016). Radiation oncology in the era of precision medicine. Nat Rev Cancer.

[2] Dawson L.A., Sharpe M.B. (2006). Image-guided radiotherapy: rationale, benefits, and limitations. Lancet Oncol.

[3] De-Colle C., Kirby A., Russell N., Shaitelman S.F., Currey A., Donovan E. (2023). Adaptive radiotherapy for breast cancer. Clin Transl Radiat Oncol.

[4] Den R.B., Doemer A., Kubicek G., Bednarz G., Galvin J.M., Keane W.M. (2010). Daily image guidance with cone-beam computed tomography for head-and-neck cancer intensity-modulated radiotherapy: a prospective study. Int J Radiat Oncol Biol Phys.

[5] Thorwarth D., Low D.A. (2021). Technical challenges of real-time adaptive MR-guided radiotherapy. Front Oncol.

[6] Yan D., Vicini F., Wong J., Martinez A. (1997). Adaptive radiation therapy. Phys Med Biol.

[7] Mittauer K.E., Hill P.M., Geurts M.W., De Costa A.M., Kimple R.J., Bassetti M.F. (2019). STAT-ART: the promise and practice of a rapid palliative single session of MR-guided online adaptive radiotherapy (ART). Front Oncol.

[8] Nelissen K.J., Versteijne E., Senan S., Hoffmans D., Slotman B.J., Verbakel W.F.A.R. (2023). Evaluation of a workflow for cone-beam CT-guided online adaptive palliative radiotherapy planned using diagnostic CT scans. J Appl Clin Med Phys.

[9] Schiff J.P., Zhao T., Huang Y., Sun B., Hugo G.D., Spraker M.B. (2023). Simulation-free radiation therapy: an emerging form of treatment planning to expedite plan generation for patients receiving palliative radiation therapy. Adv Radiat Oncol.

[10] Nelissen K.J., Versteijne E., Senan S., Rijksen B., Admiraal M., Visser J. (2023). Same-day adaptive palliative radiotherapy without prior CT simulation: Early outcomes in the FAST-METS study. Radiother Oncol.

[11] Schiff J.P., Maraghechi B., Chin R.-I., Price A., Laugeman E., Rudra S. (2023). A pilot study of same-day MRI-only simulation and treatment with MR-guided adaptive palliative radiotherapy (MAP-RT). Clin Transl Radiat Oncol.

[12] Price A.T., Schiff J.P., Silberstein A., Beckert R., Zhao T., Hugo G.D. (2024). Feasibility of simulation free abdominal stereotactic adaptive radiotherapy using an expedited pre-plan workflow. Phys Imaging Radiat Oncol.

[13] Chen L., Liang X., Shen C., Jiang S., Wang J. (2020). Synthetic CT generation from CBCT images via deep learning. Med Phys.

[14] Hooshangnejad H., Chen Q., Feng X., Zhang R., Ding K. (2023). deepPERFECT: Novel deep learning CT synthesis method for expeditious pancreatic cancer radiotherapy. Cancers (Basel).

[15] O'Hara C.J., Bird D., Al-Qaisieh B., Speight R. (2022). Assessment of CBCT–based synthetic CT generation accuracy for adaptive radiotherapy planning. J Appl Clin Med Phys.

[16] Coric I., Shreshtha K., Roque T., Paragios N., Gani C., Zips D. (2022). Dosimetric evaluation of dose calculation uncertainties for MR-only approaches in prostate MR-guided radiotherapy. Front Phys.

[17] Carr M.E., Jelen U., Picton M., Batumalai V., Crawford D., Peng V. (2023). Towards simulation-free MR-linac treatment: utilizing male pelvis PSMA-PET/CT and population-based electron density assignments. Phys Med Biol.

[18] Glober G., Holmes T.W., Chauhan B., Shah A.P., Dvorak T., Rineer J.M. (2018). A method to reduce time to start for patients receiving palliative radiation therapy for painful spine metastases. Int J Radiat Oncol Biol Phys.

[19] Glober G., Kubli A., Kielbasa J., Chauhan B., Burch D., Holmes T. (2020). Technical report: diagnostic scan-based planning (DSBP), a method to improve the speed and safety of radiation therapy for the treatment of critically Ill patients. Pract Radiat Oncol.

[20] Herrmann T., Baumann M. (2005). Die Verlängerung der Wartezeit oder der Gesamtbehandlungszeit durch ungeplante Bestrahlungspausen. Strahlenther Onkol.

[21] Pechačová Z., Lohynská R. (2021). Clinical application of time factor principles in radiotherapy in compensation of radiation series interruptions. Klin Onkol.

[22] Wong S., Roderick S., Kejda A., Atyeo J., Grimberg K., Porter B. (2021). Diagnostic computed tomography enabled planning for palliative radiation therapy: removing the need for a planning computed tomography scan. Pract Radiat Oncol.

[23] Nierer L., Walter F., Niyazi M., Shpani R., Landry G., Marschner S. (2020). Radiotherapy in oncological emergencies: fast-track treatment planning. Radiat Oncol.

[24] Schuler T., Back M., Hruby G., Carroll S., Jayamanne D., Kneebone A. (2021). Introducing computed tomography simulation-free and electronic patient-reported outcomes-monitored palliative radiation therapy into routine care: clinical outcomes and implementation experience. Adv Radiat Oncol.

[25] Uchinami Y., Kanehira T., Fujita Y., Miyamoto N., Yokokawa K., Koizumi F. (2023). Evaluation of short-term gastrointestinal motion and its impact on dosimetric parameters in stereotactic body radiation therapy for pancreatic cancer. Clin Transl Radiat Oncol.

[26] de Leon J., Jelen U., Carr M., Crawford D., Picton M., Tran C. (2024). Adapting outside the box: Simulation-free MR-guided stereotactic ablative radiotherapy for prostate cancer. Radiother Oncol.

[27] Fakhoury K.R., Schubert L.K., Coyne M.D., Aldridge W., Zeiler S., Stuhr K. (2022). Practical Implementation of Emergent After-Hours Radiation Treatment Process Using Remote Treatment Planning on Optimized Diagnostic CT Scans. Cureus.

[28] Kejda A., Quinn A., Wong S., Lowe T., Fent I., Gargett M. (2023). Evaluation of the clinical feasibility of cone-beam computed tomography guided online adaption for simulation-free palliative radiotherapy. Phys Imaging Radiat Oncol.

[29] Gospodarowicz M.K., Wittekind C., Brierley J.D. (2016).

