# Supplementary material for: Simulation-free magnetic resonance-guided radiation therapy of prostate cancer
Source: Phys Imaging Radiat Oncol. 2024 Nov 7;32:100667. doi: 10.1016/j.phro.2024.100667 (PMC11609320; doi:10.1016/j.phro.2024.100667)
Supplement: Supplementary Data 1 [file mmc1.pdf]

Supplementary material

Supplementary Table 1

Tabular overview of the number of segments in reference and adapted plans for each patient, comparing the standard and simulation-free workflows.

Patient	Standard Reference	Simulation-free Reference	Standard Adapted	Simulation-free Adapted
#1	69	67	68	68
#2	72	69	67	70
#3	64	67	66	67
#4	54	68	78	68
#5	63	66	67	70
#6	69	69	68	69
#7	78	67	79	68
#8	50	68	58	68
#9	65	69	69	68
#10	64	66	81	69

Supplementary Table 2

Overview of monitor units from reference and adapted plans, broken down by patient, of the standard and simulation-free workflows.

Patient	Standard Reference	Simulation-free Reference	Standard Adapted	Simulation-free Adapted
#1	1098	1187	1176	1155
#2	1188	1078	1252	1171
#3	969	956	980	1053
#4	941	1198	1072	1161
#5	986	1014	1034	1046
#6	1176	1192	1202	1218
#7	1204	1141	1289	1124
#8	880	1068	1008	1017
#9	1036	1125	1296	1250
#10	1059	1010	1049	1133
